# Supplementary material for: Direct detection of SARS-CoV-2 using non-commercial RT-LAMP reagents on heat-inactivated samples
Source: Sci Rep. 2021 Jan 19;11:1820. doi: 10.1038/s41598-020-80352-8 (PMC7815738; doi:10.1038/s41598-020-80352-8)
Supplement: Supplementary file 1 — Supplementary Information. [file 41598_2020_80352_MOESM1_ESM.docx]

**Supplementary Information**

**Direct detection of SARS-CoV-2 using non-commercial RT-LAMP reagents on heat-inactivated samples.**

Alisa Alekseenko ^1^*, Donal Barrett ^1^*, Yerma Pareja-Sanchez ^1^*, Rebecca J Howard ^2^, Emilia Strandback ^3^, Henry Ampah-Korsah ^3^, Urška Rovšnik ^2^, Silvia Zuniga-Veliz ^4^ , Alexander Klenov ^5^, Jayshna Malloo ^5^, Shenglong Ye ^6^, Xiyang Liu ^6^ , Björn Reinius ^3^, Simon J Elsässer ^7^, Tomas Nyman ^3^, Gustaf Sandh ^4^ , Xiushan Yin ^1,6,8^, Vicent Pelechano ^1#^

^1^ SciLifeLab, Department of Microbiology, Tumor and Cell Biology. Karolinska Institutet, Solna, Sweden.

^2^ SciLifeLab, Department of Biochemistry and Biophysics, Stockholm University, 17121 Solna, Sweden

^3^ Department of Medical Biochemistry and Biophysics, Karolinska Institutet, Solna, Sweden.

^4^ Department of Clinical Microbiology, Karolinska University Hospital, Stockholm, Sweden.

^5^ Hudak Lab, Department of Biology, York University, 4700 Keele St, Toronto, ON, M3J 1P3, Canada

^6^ Biotech & Biomedicine Science (Shenyang）Co. Ltd, Shenyang, 110000, China

^7^ SciLifeLab, Department of Medical Biochemistry and Biophysics, Division of Genome Biology, Karolinska Institutet, Solna, Sweden.

^8^ Applied Biology Laboratory, Shenyang University of Chemical Technology, 110142, Shenyang, China

* Co-first authors with equal contribution to this work.

^#^ Correspondence may be addressed to Vicent Pelechano ([vicente.pelechano.garcia@ki.se](mailto:vicente.pelechano.garcia@ki.se), phone: +46728564904, mailing address: Tomtebodavägen 23A, 17165 Solna, Sweden).

**Supplementary Figure 1**

**
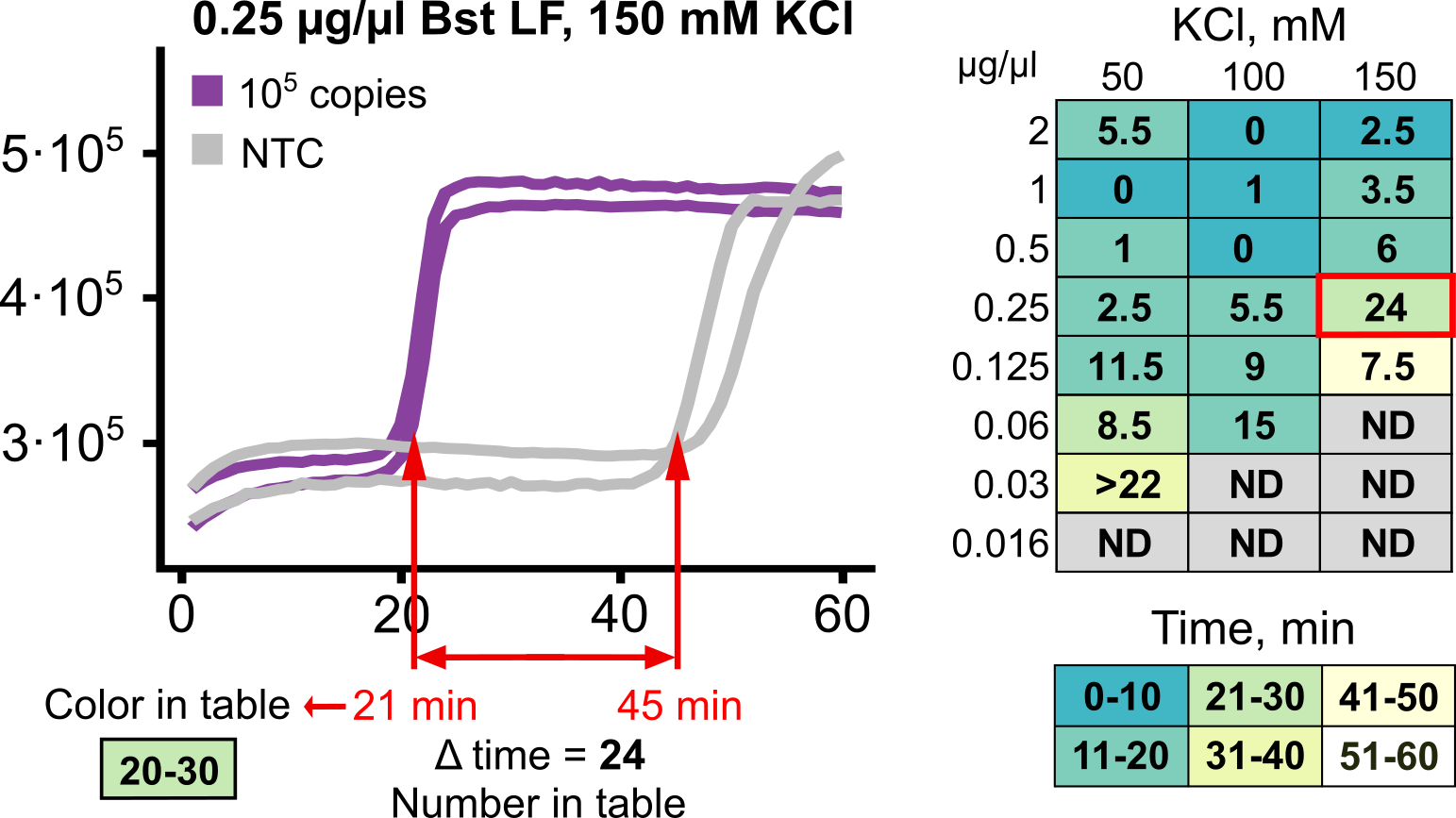
**

**Figure S1. A graphical example of the method we used to construct tables in Fig. 1B, 2C, and S3A-D.** We performed RT-LAMP with duplicates of positive control (synthetic template) and negative control (no template) at a range of enzyme concentrations and noted down the time at which the amplification curves reached exponential phase. We wanted to select the condition with the best separation of positive and negative control curves and the fastest amplification of the positive control. Therefore, we noted down two values: 1. the time difference between the **slowest** positive control and the **fastest** negative control; 2. the time to exponential phase for the **slowest** positive control. In the tables, the first set of values is repesented as **numbers** and the second set of values as **colors**, depending on the range they fall into (0-10, 11-20, 21-30, 31-40, 41-50, or 51-60 minutes). “>...” indicates that the negative control did not amplify within 1 hour. ND indicates no amplification of either positive or negative control. NA indicates that the condition was not tested.

**Supplementary Figure 2**

**
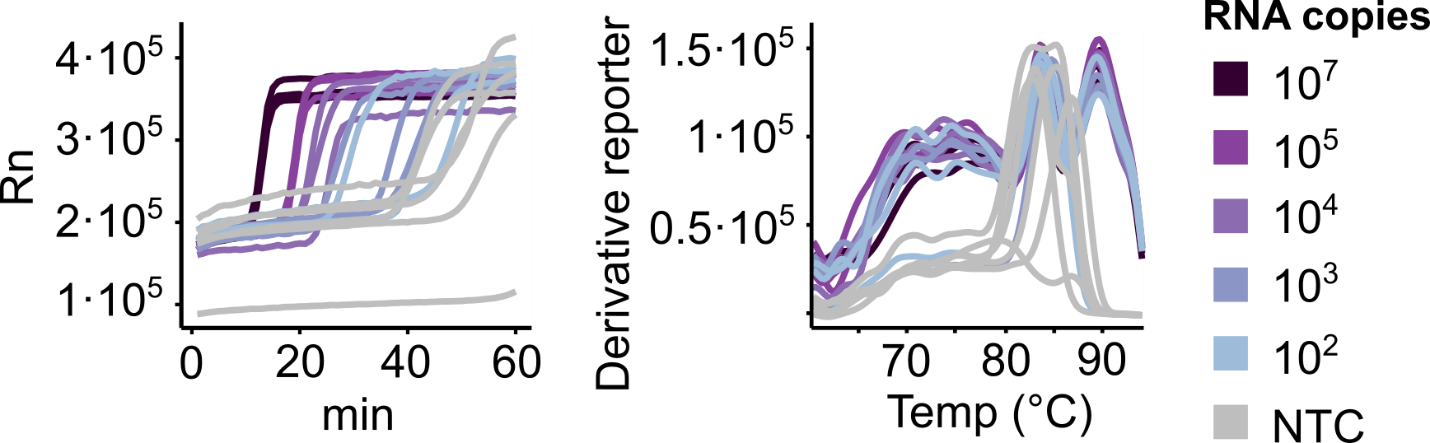
**

**Figure S2. An example of melting curve analysis.** RT-LAMP was performed with 0.06 µg/µl v5.9 in ThermoPol buffer with different amount of synthetic template (three replicates each) and NTCs (six replicates). Amplification curves are plotted on the left. After 60 minutes at 65 °C, we performed a melting curve analysis with steps of 0.3 °C between 60 and 95 °C. The derivative reporter values as calculated by the StepOne software are presented on the right. All NTC reactions had different profiles than the majority of the positive controls, demonstrating that the amplification was a result of non-specific primer interactions and not presence of contaminating template. However, two of the positive control reactions (one with 10^2^ copies of RNA and another with 10^3^ copies) demonstrated profiles similar to the NTCs. This indicates that with low template amounts, sometimes the spurious primer-templated amplification outcompetes the specific amplification.

**Supplementary Figure 3**


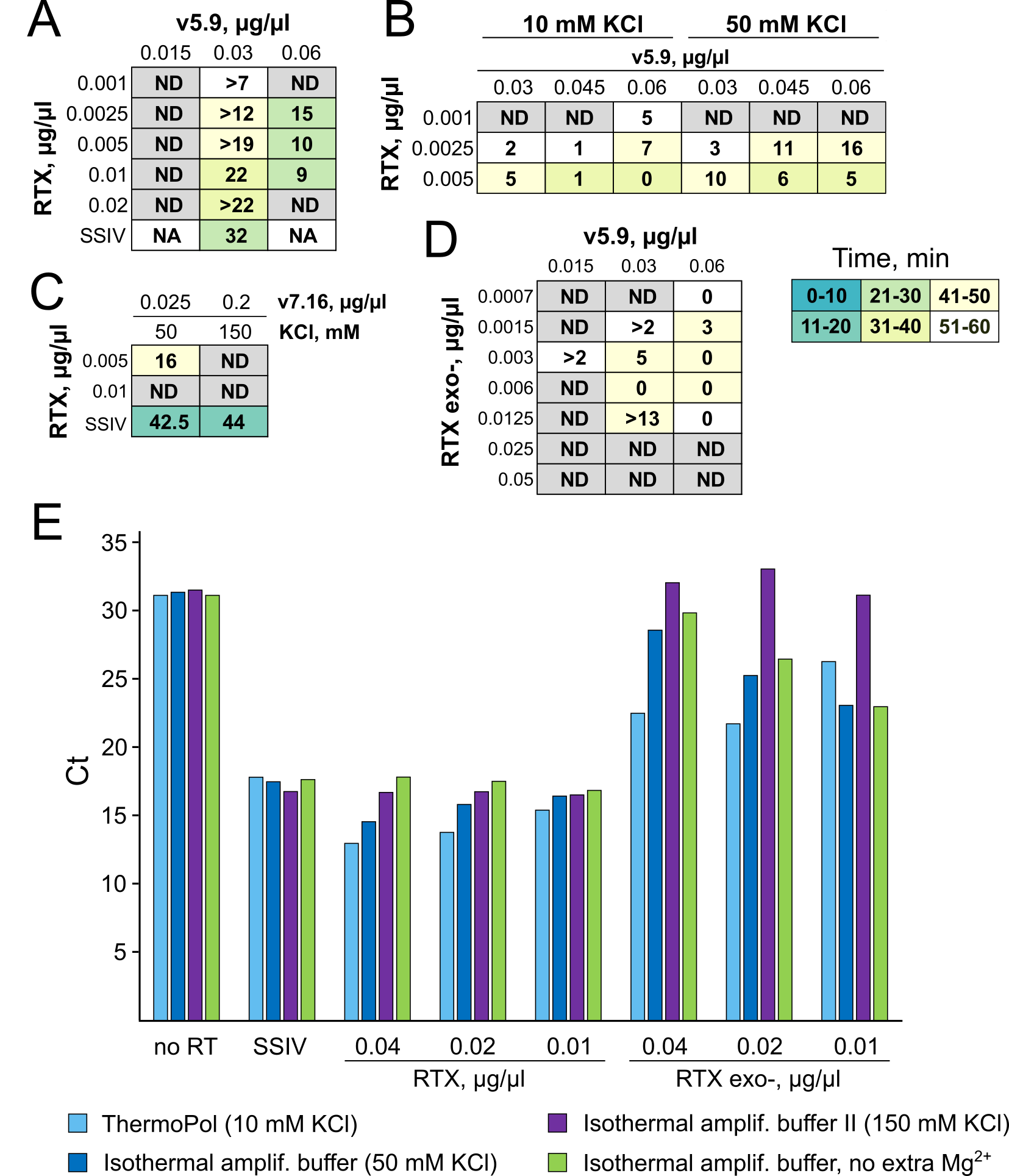


**Figure S3. Analysis of RTX performance.** Tables showing the performance of RTX together with either v5.9 or v7.16 at different concentrations of the enzymes and KCl. The numbers and colors were determined in the same way as in Figures 1 and 2 (see Fig. S1 for explanation). **A.** 83000 copies of synthetic RNA. **B.** 8300 copies. **C**. 1000 copies. **D.** Table showing performance of RTX^exo-^ together with v5.9 at different enzyme concentrations (8300 copies of synthetic RNA). **E.** Results of RT-qPCR demonstrate that RTX and RTX^exo-^ do possess RT activity, as compared to SSIV (see methods). However, RTX^exo-^ has poor performance at higher KCl concentrations.

**Supplementary Figure 4**

**Figure S4. Dependence of Bst3.0 on additional RT activity.** Amplification of As1e and iLACO synthetic templates with the corresponding primers using Bst3.0 without an RT enzyme. (Same experiment as Figure 3.)

**Supplementary Table 1.**

Contains the raw data for the RT-LAMP experiments shown as amplification curves in the figures.

**Supplementary Table 2.**

Contains all the Ct values for clinical samples from GeneXpert and all the RT-LAMP trials.
